# Extension of the *Caenorhabditis elegans* Pharyngeal M1 Neuron Axon Is Regulated by Multiple Mechanisms

**DOI:** 10.1534/g3.113.008466

**Published:** 2013-11-01

**Authors:** Osama Refai, Patricia Rohs, Paul E. Mains, Jeb Gaudet

**Affiliations:** Department of Biochemistry and Molecular Biology, Alberta Children’s Hospital Research Institute, University of Calgary, Calgary, Alberta T2N 4N1, Canada

**Keywords:** *Caenorhabditis elegans*, neuron, axon, genetics, pharynx

## Abstract

The guidance of axons to their correct targets is a critical step in development. The *C. elegans* pharynx presents an attractive system to study neuronal pathfinding in the context of a developing organ. The worm pharynx contains relatively few cells and cell types, but each cell has a known lineage and stereotyped developmental patterns. We found that extension of the M1 pharyngeal axon, which spans the entire length of the pharynx, occurs in two distinct phases. The first proximal phase does not require genes that function in axon extension (*unc-34*, *unc-51*, *unc-115*, and *unc-119*), whereas the second distal phase does use these genes and is guided in part by the adjacent g1P gland cell projection. *unc-34*, *unc-51*, and *unc-115* had incompletely penetrant defects and appeared to act in conjunction with the g1P cell for distal outgrowth. Only *unc-119* showed fully penetrant defects for the distal phase. Mutations affecting classical neuronal guidance cues (Netrin, Semaphorin, Slit/Robo, Ephrin) or adhesion molecules (cadherin, IgCAM) had, at best, weak effects on the M1 axon. None of the mutations we tested affected the proximal phase of M1 elongation. In a forward genetic screen, we isolated nine mutations in five genes, three of which are novel, showing defects in M1, including axon overextension, truncation, or ectopic branching. One of these mutations appeared to affect the generation or differentiation of the M1 neuron. We conclude that M1 axon extension is a robust process that is not completely dependent on any single guidance mechanism.

During development, axons navigate toward their final targets in a highly dynamic and precisely regulated process. Correct axon targeting requires the combined action of a variety of conserved signaling molecules sensed by the growth cone (Chisholm and Tessier-Lavigne 1999; Dickson 2002; Yu and Bargmann 2001). Some signaling molecules act as long-range repellents or attractants (Araujo and Tear 2003; Dickson 2002). Other signaling molecules act locally to break the distance traveled by the axon into a series of shorter trajectories, causing the axon to change direction at specific choice points. Also known as guidepost cells or intermediate targets, such choice points may be provided by neuronal support cells, non-neuronal cells, or pioneer neurons (Araujo and Tear 2003; Bate 1976; Bentley and Caudy 1983; Colamarino and Tessier-Lavigne 1995; Colon-Ramos and Shen 2008; Hidalgo 2003; Hidalgo and Booth 2000; Hutter 2003; Raper and Mason 2010).

The past several decades have witnessed great progress in the identification of mechanisms necessary for axon pathfinding, yet known pathways still cannot explain all of the complexity required to build nervous systems (O’Donnell *et al.* 2009; Schmitz *et al.* 2007). Thus, additional axon guidance mechanisms almost certainly remain to be identified. *C. elegans* has proved to be an excellent model for the discovery of key axon guidance cues and regulators, which are commonly conserved among invertebrates and vertebrates (Chisholm and Tessier-Lavigne 1999; Culotti and Merz 1998). In addition to being amenable to large-scale genetic screens, the transparent worm allows *in vivo* visualization of living neurons using fluorescently tagged proteins (Hutter 2004).

The *C. elegans* pharynx has a very simple nervous system that consists of only 20 neurons, each with a stereotyped trajectory. This system develops independently from the rest of the nervous system and thus may use unique pathfinding mechanisms. Neurons elsewhere in the body usually grow between basement membranes laid down by epidermis and muscles. In contrast, pharyngeal neurons lie within the pharyngeal muscle folds (Albertson and Thomson 1976; Avery and Thomas 1997; Graham *et al.* 1997; Pilon and Morck 2005). The pharyngeal nervous system is organized into one dorsal nerve cord, two subventral nerve cords, and a pharyngeal nerve ring. Because individual ablation of most of these neurons has no visible effect, pharyngeal neurons are thought to act redundantly to regulate the rhythmic contractions of the pharynx (Avery 1993; Avery and Horvitz 1989; Avery and Thomas 1997; Pilon and Morck 2005). Therefore, severe defects in many pharyngeal neurons should be viable, an advantage for analysis of mutant phenotypes.

The unique morphology of each of the 20 pharyngeal neurons raises the possibility that different neurons may use distinct mechanisms to establish their trajectories. Previous analysis of the axon guidance of two left/right pairs of pharyngeal neurons, NSML/NSMR and M2L/M2R, revealed that they use known guidance mechanisms, such as Netrin and Slit/Robo, and genes such as *unc-51* and *unc-119*, but in different ways from each other (Axang *et al.* 2008; Morck *et al.* 2003; Pilon 2008). The genetic requirements differ between the different processes of the NSM neurons (Axang *et al.* 2008). As with other examples of axon guidance in *C. elegans*, the extension of the NSM and M2 axons is robust, with mutations in genes with guidance cue and growth cone functions generally having incomplete penetrance.

The NSM and M2 pharyngeal neurons also use unusual growth cone–independent mechanisms (Axang *et al.* 2008; Morck *et al.* 2003; Pilon 2008), extending for part of their journeys using the “fishing line” paradigm (Bray 1984; Chada *et al.* 1997; Heidemann *et al.* 1995; Zheng *et al.* 1991). In this model, the neuron soma (“the fish”) may pull out the axon (“the line”) as the cell body moves away from an initial attachment point. Alternatively, the cell body may remain stationary with the axon drawn out by an attachment to a migrating neighbor (Bray 1984; Pilon 2008). This concept was demonstrated experimentally by applying mechanical tension to the short axonal processes of cultured neurons. In response to mechanical stimuli, rat embryonic dorsal root ganglia neurons increase their length and rate of growth (Loverde *et al.* 2011; Pfister *et al.* 2006; Smith 2009). *Drosophila* larval optic nerves, and possibly the cerebellar granule cells in mammals, use similar mechanisms to extend their axons (Jan *et al.* 1985; Komuro and Yacubova 2003; Yacubova and Komuro 2003). In *C. elegans*, morphogenesis of the pharynx as a whole is thought to provide the motive force for the fishing line to draw out the M2 and NSM axons (Morck *et al.* 2006; Morck *et al.* 2003; Morck *et al.* 2004; Pilon 2008). Amphid dendrites also extend by a similar mechanism (Heiman and Shaham 2009).

Here, we explore axon guidance in the *C. elegans* pharynx by analyzing axon extension of the pharyngeal motor neuron M1. Unlike the previously studied pharyngeal neurons, the M1 axon spans nearly the whole length of the pharynx, navigating through several different pharyngeal compartments (Albertson and Thomson 1976). Similar to the M2 and NSM neurons, M1 may use growth cone–independent mechanisms to build the initial part of its trajectory. Additionally, we found that M1 relies partially on the adjacent non-neuronal g1P gland cell to navigate the distal portion of its trajectory using genes implicated in growth cone function. Unlike the M2 and NSM neurons, the M1 axon is not affected by mutations in the Netrin and Sax/Robo guidance pathways, but it uses the Semaphorin system to a small extent. However, phenotypes for mutations affecting axon outgrowth and guidance are incompletely penetrant, indicating redundant mechanisms likely mediate M1 axon extension. Finally, a genetic screen for M1-defective phenotypes identified nine mutations for M1 development, including a mutation that may affect neuronal differentiation or cell fate specification. We suggest that the M1 system provides an important new model for the study of axon guidance.

## Materials and Methods

### Nematode strains and culture

*C. elegans var*. Bristol was used as the reference wild-type (Brenner 1974). Standard methods for the culture, manipulation, and genetics of *C. elegans* were used (Sulston and Hodgkin 1988). All strains were cultured at 20° unless otherwise stated. The following mutations were used. Alleles with an “*iv*” designation were derived in this study. Descriptions of all others are found at www.wormbase.org. Linkage group (LG) I contained *lin-17(n3091)*, *mab-20(bx24)*, *smp-1(ev715)*, *smp-2(ev709)*, *unc-40(e271)*, *unc-73(e936)*, *rig-5(hd48)*, *pry-1(mu38)*, and *mnm-8(iv82)*. LG II contained *vab-1(dx31* and *e2)*, *rig-6(gk376)*, and *plx-2(ev773)*. LG III contained *unc-69(e587)*, *unc-119(e2498)*, *mnm-7(iv77* and *iv90)*, and *cdh-4(ok1323)*. LG IV contained *unc-5(e53)*, *unc-129(ev554)*, *rig-4(hd47)*, *mnm-6(iv88)*, *egl-20(hu105)*, *plx-1(ev724)*, and *ced-3(n717)*. LG V contained *vab-8(ct33) dpy-11(e224)*, *unc-34(e315)*, *unc-51(e369) dpy-21(e428)*, *unc-51(iv84)*, *him-5(e1490)*, *unc-76(e911)*, *rpm-1(js317*, *ur299*, *iv78*, *iv79*, *iv80*, and *iv81)*, *fmi-1(rh308)*, and *ced-10(n1993)*. LG X contained *sax-3(ky123)*, *slt-1(eh15)*, *unc-6(ev400* and *e78)*, *unc-115(e2225)*, *rig-1(hd15)*, *wrk-1(hd45)*, *rig-3(hd51)*, *(hd18)*, *ncam-1(hd49)*, *mig-2(mu28)*, and *egl-15(n484)*. The *mab-20(bx24)*, *smp-1(ev715)*, *smp-2(ev709)*, *plx-1(ev724)*, *plx-2(ev773)*, and *smp-1(ev715)*; *smp-2(ev709)* strains included *him-5(e1490)*. The *smp-1(ev715)*; *smp-2(ev709)* strain was generously provided by J. Culotti (Ginzburg *et al.* 2002). The octuple mutant *rig-5*; *rig-6*; *rig-4*; *rig-1wrk-1rig-3syg-1ncam-1* was a kind gift from H. Hutter (Schwarz *et al.* 2009), and *rpm-1(ur299)* was kindly provided by W.G. Wadsworth (Li *et al.* 2008).

In all of our experiments, the five pharyngeal gland cells, including g1P, were visualized using the gland markers *hlh-6*::*yfp*, *hlh-6*::*mTomato* (Smit *et al.* 2008) or *phat-1*::*wCherry*, which is a worm-optimized form of mCherry. The integrated worm transgene *ivIs26[phat-1*::*wCherry glr-2*::*gfp pRF4(rol-6(su1006))]I* and the following transgenic lines were developed for this work: *ivEx136[glr-2*::*gfp hlh-6*::*mTomato rol-6(su1006)]*, *ivEx138[hlh-6*::*egl-1 glr-2*::*gfp rol-6(su1006)]*, *ivEx139[ceh-2*::*egl-1 glr-2*::*gfp elt-2*::*mTomato rol-6(su1006)]*, *ivEx140[ceh-2*::*egl-1 ceh-2*::*gfp elt-2*::*mTomato rol-6(su1006)]*, *ivEx143[hlh-6*::*egl-1 ceh-2*::*egl-1 elt-2*::*mTomato]*, *Ex144[ceh-2*::*gfp elt-2*::*mTomato]*, *ivEx272[glr-2*::*gfp rol-6(su1006)]*, *ivEx273[glr-2*::*wCherry rol-6(su1006)]*, *ivEx366[hlh-6*::*egl-1 hlh-6*::*yfp elt-2*::*mCherry rol-6(su1006)]*, *ivEx387[phat-1*::*wCherry glr-2*::*gfp pRF4(rol-6(su1006))]*. Chi-square was used to calculate the p-values to test for statistical significance between strains.

### Construction of transgenic lines

All reporters were made by PCR amplification of promoter fragments from genomic DNA, followed by cloning into pPD95.77, pPD95.77-YFP (gifts from A. Fire), or pJH1774 (a gift from M. Zhen), which contain the coding sequence for *gfp*, *yfp* or *wCherry*, respectively. The M1 reporters were constructed by PCR amplification of the *glr-2* promoter (1.7 kb upstream of the ATG), followed by cloning into pPD95.77 or pJH1774. Similarly, I3 reporter was constructed by PCR amplification of the *ceh-2* promoter (1.6 kb upstream of the ATG), followed by cloning into pPD95.77.

Transformation was performed as described by (Mello and Fire 1995). DNA was injected at 20–30 ng/mL together with 50 ng/mL pRF4 *(rol-6(su1006))*, which confers a dominant Roller phenotype (Mello *et al.* 1991) and sufficient pBS II (SK+) to bring the total DNA concentration to 100 ng/mL. For some analyses, we included 20 ng/mL of an intestine-specific reporter, *elt-2*::*mTomato*::*HIS2B* (Fukushige *et al.* 1998), which served as an independent marker for transgenic arrays. Extrachromosomal arrays were integrated using 3000 R of gamma irradiation following the standard methods and were outcrossed five times to N2.

### Cell ablation

To induce gland cell death, the *hlh-6::egl-1* construct was injected at 20 ng/mL with 20 ng/mL elt-2::mTomato::HIS2B, 30 ng/mL pBS II (SK+) and 20 ng/mL *hlh-6::yfp* as described (Smit *et al.* 2008). Transgenic (*i.e.*, *elt-2::mTomato::HIS2B* expressing) embryos were collected at different times to analyze the efficiency and timing of the gland cell death as assessed by YFP expression. Embryos were mounted on 2% agarose pads and scored with a Zeiss Axioplan compound microscope. I3 ablation was induced using the ce*h-2*::*egl-1* construct, which was injected at 20 ng/mL with 20 ng/mL *elt-2*::*mTomato*::*HIS2B*, 30 ng/mL pBS II (SK+) and 20 ng/mL *ceh-2*::*gfp*. *elt-2*::*mTomato*–positive L4 and young adult transgenics were scored under the fluorescent microscope to determine I3 ablation efficiency.

### Screen for M1 neuron defects mutants

EMS mutagenesis was performed according to the standard protocol (Sulston and Hodgkin 1988). Mutagenized *ivIs26[phat-1*::*wCherry glr-2*::*gfp pRF4(rol-6(su1006)]* hermaphrodites, which carry reporters for the M1 neuron and gland cells, were cultured on 9-cm plates in groups of five at 20°. Four days later, L3 and L4 F1 progeny were placed individually on 35-mm plates and cultured at 15° or 20°. Between 6 and 9 days later, F2 or F3 L1 progeny were screened under a fluorescence dissecting microscope for individuals with abnormal M1 neurons. Mutations were outcrossed at least five times to wild-type. Mutants were mounted on 2% agarose pads, paralyzed with 100 mM levamisole, and scored with a Zeiss Axioplan compound microscope for detailed analysis. Unless stated otherwise, we scored defects in newly hatched L1 larva.

### Mapping of mutations

Single nucleotide polymorphism (SNP) mapping was performed as described by Wicks *et al.* (2001) and Davis *et al.* (2005). The *ivIs26[phat-1*::*wCherry glr-2*::*gfp pRF4(rol-6(su1006)]* bearing mutants were crossed with Hawaiian strain (CB4856) males. For each mutation, 50 M1 defective F2 segregants and fifty M1 normal F2 animals were separately pooled and lysed following standard protocols (Davis *et al.* 2005). The mutant and nonmutant lysates were then separately used as DNA templates for PCR amplification as described (Davis *et al.* 2005). We used primer pairs for SNP markers described at genome.wustl.edu or by Davis *et al.* (2005). Mutations that mapped to the same chromosome and had similar phenotypes were tested for complementation.

## Results

### The *glr-2::gfp* allows visualization of the M1 neuron

**Figure 1:**
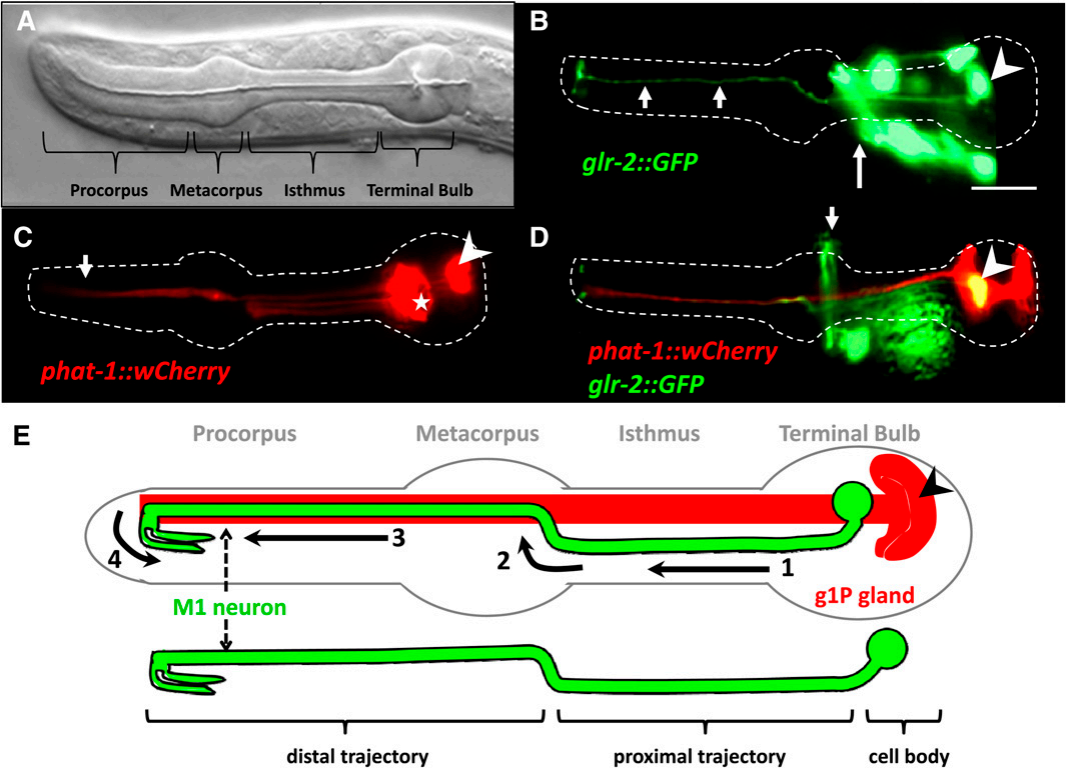
Anatomy of the pharynx showing the M1 neuron and the g1P gland in L4 or young adult. (A) Nomarski image of the *C. elegans* pharynx. (B) Fluorescence micrographs of M1 marked by the *glr-2*::*gfp* reporter (arrowhead). Other nonpharyngeal green cells are part of the nerve ring (long arrow) and can be distinguished from M1 by position. Note the M1 projection into the procorpus (short arrows). (C) Fluorescence micrographs of the pharyngeal gland cells marked by the *phat-1*::*wCherry* reporter. The g1P cell body (arrowhead) is located in the terminal bulb and extends a projection to the procorpus (arrow). Other gland cells are also marked with this reporter (★). (D) Double transgenic animals with a gland-expressed *phat-1*::*wCherry* and the *glr-2*::*gfp*. Arrowhead indicates the M1 cell body and the arrow denotes the nonpharyngeal cells in the nerve ring. (E) Diagram of the *C. elegans* pharynx (outlined in gray) with the M1 neuron (green) and g1P gland (red). The different portions of the M1 axon trajectory are numbered from 1 through 4 (see text). Modified from (Albertson and Thomson 1976). Scale bar in (B) = 10 µm.

The four anatomical structures of the pharynx are, from anterior to posterior, the procorpus, metacorpus, isthmus, and terminal bulb (Figure 1A). Each of the 20 *C. elegans* pharyngeal neurons has a unique morphology. We chose to focus on the M1 neuron, which has its cell body located in the terminal bulb. Electron microscopy reconstructions indicate that the M1 axon has four components to its trajectory, as outlined on Figure 1E (Albertson and Thomson 1976). First, the M1 axon extends anteriorly from its cell body in the terminal bulb through the isthmus to the metacorpus (the “proximal trajectory”). Second, when the M1 axon passes the pharyngeal nerve ring in the metacorpus, it deflects dorsally to join the dorsal nerve cord and contacts the projection of the g1P gland. Third, the M1 axon runs anteriorly along the g1P gland projection (the “distal trajectory”). Near the anterior end of the procorpus, the M1 axon splits into two projections, which synapse on the anterior-most pharyngeal muscles, pm1 and pm2. Fourth, the M1 axon reflexes ventrally and posteriorly to reach the two subventral pharyngeal nerve cords.

To monitor M1 axon migration, we used the marker *glr-2*::*gfp* that is expressed in M1 motor neuron (Figure 1B). This transgene is also expressed in a number of neurons outside the pharynx, in the nerve ring, and in the ventral nerve cord. These cells are easily distinguished from M1 based on position (Aronoff *et al.* 2004; Brockie *et al.* 2001). M1 is born at ∼330 min after fertilization (Sulston *et al.* 1983). The *glr-2*::*gfp* expression was first observed at 550 min (Brockie *et al.* 2001), when the developing embryo has lengthened to three-times that of the eggshell (three- fold stage) (Chisholm and Hardin 2005). The M1 axon is fully extended by this time; therefore, we cannot use this marker to directly observe M1 axon migration before the three-fold stage (the early pan-neuronal markers *rgef-1*::*gfp*) (Altun-Gultekin *et al.* 2001) or *unc-119*::*gfp* (Knobel *et al.* 2001; Maduro and Pilgrim 1995) were not useful because we could not distinguish M1 from other pharyngeal neurons, even when the adjacent g1P gland cell was also marked.

Like M1, the gland g1P cell body is located in the terminal bulb and sends a projection anteriorly, within the pharyngeal nerve cord, and terminates near the anterior limit of the pharynx (Figure 1C) (Smit *et al.* 2008). Using *phat-1*::*wCherry*, *hlh-6*::*yfp* or *hlh-6*::*mTomato* to mark g1P (Smit *et al.* 2008), we observed that the distal trajectory of the M1 axon extends along the g1P projection (Figure 1, B–D) as previously noted by electron microscopy (Albertson and Thomson 1976).

### Genetic ablation of the g1P pharyngeal gland cell affects the distal, but not the proximal, trajectory of the M1 axon

**Figure 2:**
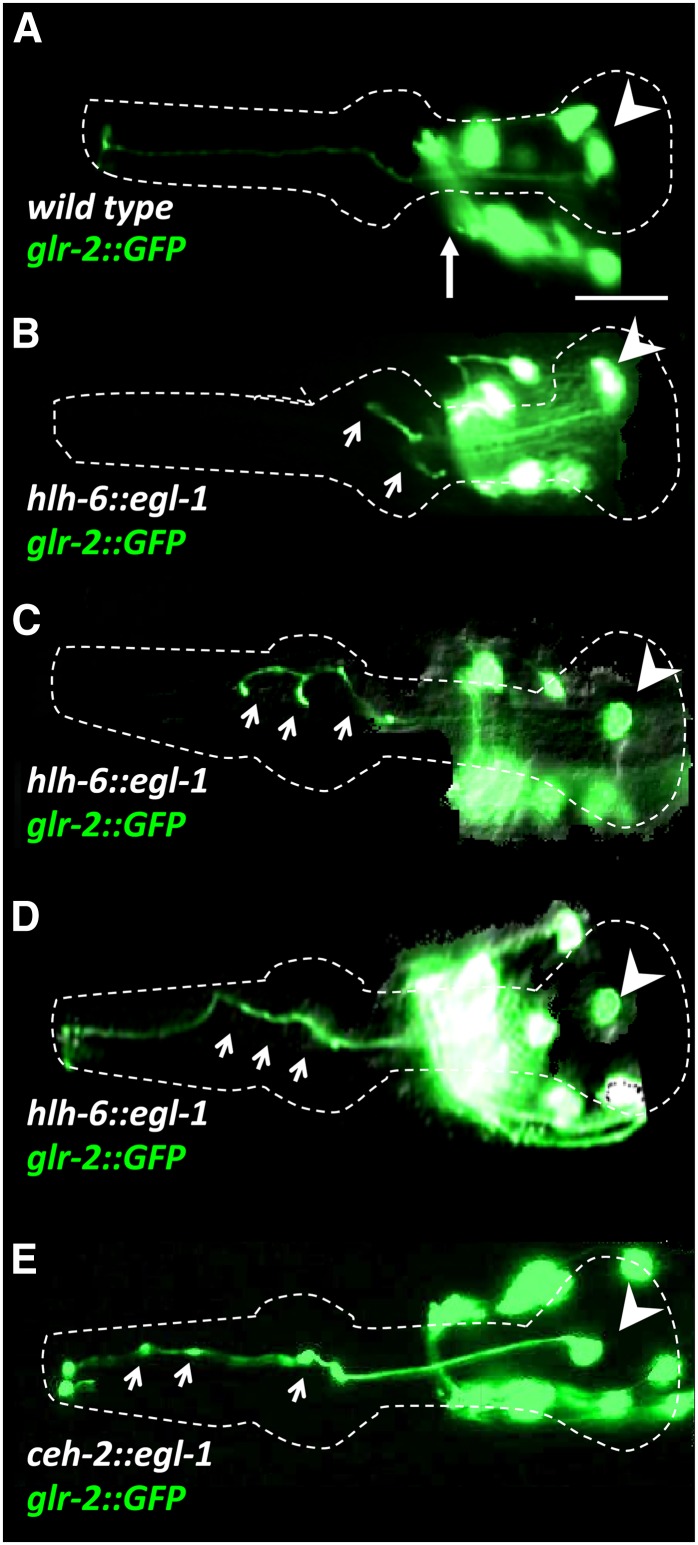
M1 axon phenotypes (marked with *glr-2*::*gfp)* in wild-type (A) and gland-ablated *hlh-6*::*egl-1* (B–D) or in I3 neuron-ablated *ceh-2*::*egl-1* transgenics (E). Arrowhead denotes the M1 cell body and the long arrow in wild-type (A) indicates the nonpharyngeal cells in the nerve ring in these at L4 or young adults. Short arrows highlight M1 defects. (B–D) In 56% of gland-ablated transgenics, the M1 axon is either truncated (B) with abnormal branching in the metacorpus and procorpus (C) or follows an abnormal trajectory in the procorpus (D). (E) In 7% of I3 ablated animals, the M1 axon in the procorpus exhibits an abnormal shape with GFP swelling (arrows), although the axon always extended fully. Scale bar in (A) = 10 µm.

**Figure 3:**
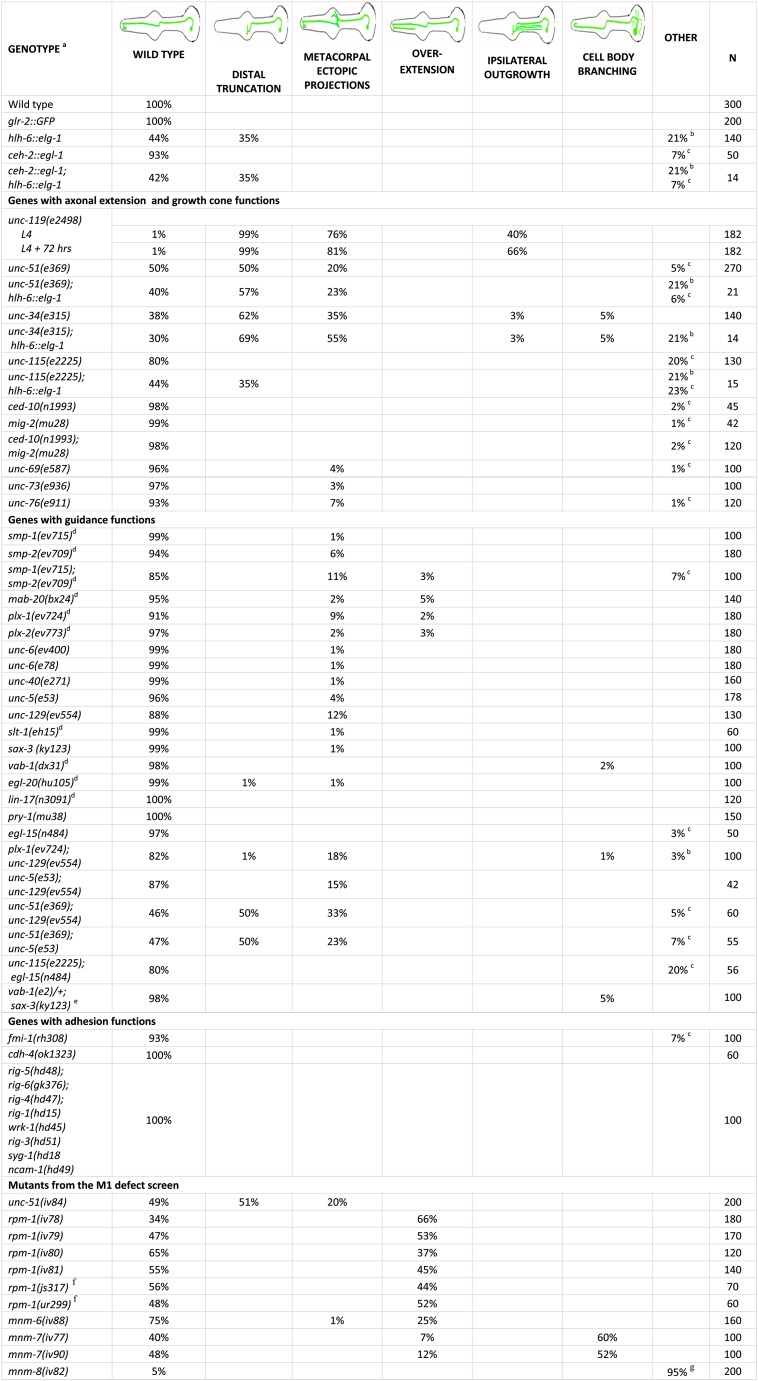
Quantification of M1 axon phenotypes are shown for mutants or after cell ablation. All values are in percents, and for each category a cartoon showing representative examples of the axonal phenotypes is shown. Footnotes on the figure are as follows. (A) All strains included *glr-2*::*gfp*, except as noted. Some neurons had multiple abnormalities and therefore some genotypes sum to >100%. (B) M1 showed abnormal trajectories in the procorpus. (C) Axons showed swelling or small ectopic projections in the procorpus. (D) Independent *glr-2*::*gfp* transgenes were made for each of these strains. (E) This was the maternal genotype as *vab-1(e2)*; *sax-3(ky123)* homozygous segregants arrest before the stage when M1 can be scored. Note that two-thirds of scored progeny will be *vab-1/+*; *sax-3*. (F) These *rpm-1* alleles were identified independently of this screen. (G) M1 neuron was absent as scored by the absence of *glr-2*::*gfp* in the pharynx.

To examine whether the adjacent g1P gland projection is necessary for the M1 axon extension in the procorpus, we genetically ablated the gland cells. The gland-specific *hlh-6* promoter (Smit *et al.* 2008), which is first expressed at the “bean” stage (380 min), was used to drive expression of the pro-apoptotic gene *egl-1* (Conradt and Horvitz 1998) in g1P soon after its birth at ∼360 min. We found that the majority of such animals exhibited M1 axon abnormalities within the procorpus. Of gland-ablated worms (n = 140), 35% showed premature axon termination in the procorpus, frequently with extra small branches (Figure 2, B and C and Figure 3). Another 21% extended beyond the procorpus but followed abnormal trajectories (Figure 2D). Notably, the proximal trajectory of the axon from the M1 cell body to the procorpus was normal in all animals. Although other gland cells were killed, none sends projections into the metacorpus or procorpus, so they are unlikely to be involved in M1 guidance. Furthermore, no major structural defects in the other pharyngeal tissues were detected (data not shown).

**Figure 4:**
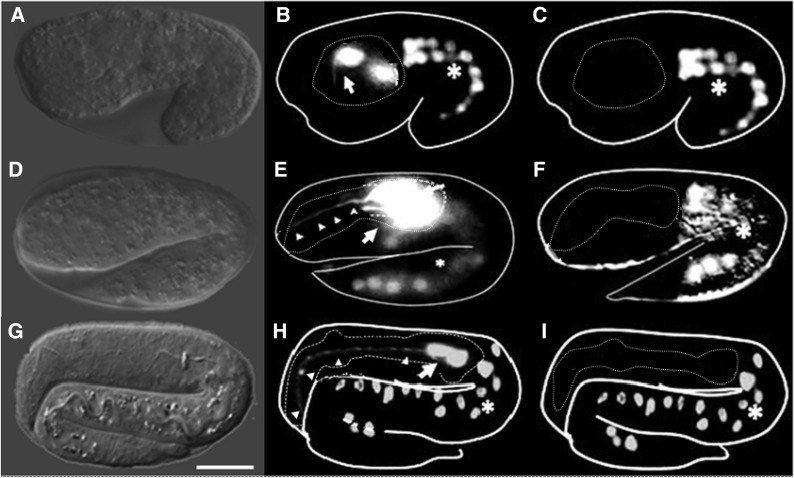
Time course measuring the efficiency and timing of the gland killing in *hlh-6*::*egl-1* transgenics. Examples of 1.5-fold (first row), two-fold (second row), and three-fold (third row) embryos are shown. First column (A, D, G) shows Nomarski images, second column (B, E, H) shows wild-type glands visualized using the *hlh-6*::*yfp* reporter, and the third column (C, F, I) represents *hlh-6*::*egl*-1 transgenics at similar developmental stages. Note the absence of the *hlh-6*::*yfp* reporter in the glandless embryos (C, F, I). Arrow indicates gland cell bodies and arrowheads indicate the g1P projection. Intestine expressing *elt-2*::*tdCherry* transgene marker (*). Scale bar in (G) = 5 µm.

The incomplete penetrance of the M1 phenotype in the gland-ablated *hlh-6*::*egl-1* transgenics could imply incomplete or delayed gland killing. Therefore, we performed a time course analysis to score killing efficiency (Figure 4). Using the *hlh-6*::*yfp* gland reporter (Smit *et al.* 2008), we observed that the g1P appeared to initiate its projection at the 1.5-fold stage, soon after the gland birth (Figure 4B) (J. Kormish and J. Gaudet, unpublished data). Additionally, we noted that the g1P projection was always fully formed by the late two-fold or early three-fold stage (∼500 min) in the normal embryos (Figure 4, E and H) (J. Kormish and J. Gaudet, unpublished data). However, only 2% of the gland-ablated embryos had normal glands (n = 70), indicating that poor transgene expression or mosaicism for the unintegrated array cannot explain the incomplete penetrance. YFP was completely absent in 38% of those embryos between 1.5-fold and three-fold (430–520 min). Residual YFP (likely remnants of apoptotic cell corpses) was apparent in the remaining 60%, but no distinct gland projection or cell bodies were observed. Therefore, the gland was efficiently killed before it developed its projection.

The fact that 44% of the gland-ablated worms apparently had a normal M1 axon implies that M1 uses more than one mechanism to extend in this region. Furthermore, the initial outgrowth of M1 is independent of the gland cell, indicating that different (or perhaps overlapping) mechanisms are used during the different phases of the journey. Thus, we genetically ablated the I3 interneuron that bundles with the M1 axon and g1P projection in the procorpus (Albertson and Thomson 1976). I3 apoptosis was induced using a transgenic array with the *ceh-2* promoter (Aspock *et al.* 2003) driving *egl-1* as well as *gfp* to mark the cell. I3 was eliminated in 97% of animals (n = 63). Using this construct, the M1 axon always reached its final target with a normal trajectory. We occasionally (7%; n = 50) observed swellings of the M1 axon in the procorpus (Figure 2E and Figure 3). Moreover, double ablation of I3 and g1P was not significantly additive (*P* < 0.05), resulting in 42% normal M1 axons (n = 14, the strain was unhealthy and difficult to maintain) compared to 44% for the g1P single ablation. These data suggest that distal M1 axon outgrowth relies on the presence of the g1P projection in the procorpus, in concert with other elements.

### The distal, but not proximal, trajectory of M1 axon is affected by mutations that impair growth cone function

To test whether M1 axon outgrowth may use a growth cone–dependent mechanism, we examined mutations with impaired growth cone and axonal extension functions. We either crossed the *glr-2*::*gfp* marker described into these backgrounds or created new extrachromosomal *glr-2*::*gfp* arrays by injection into appropriate strains (Figure 3).

**Figure 5:**
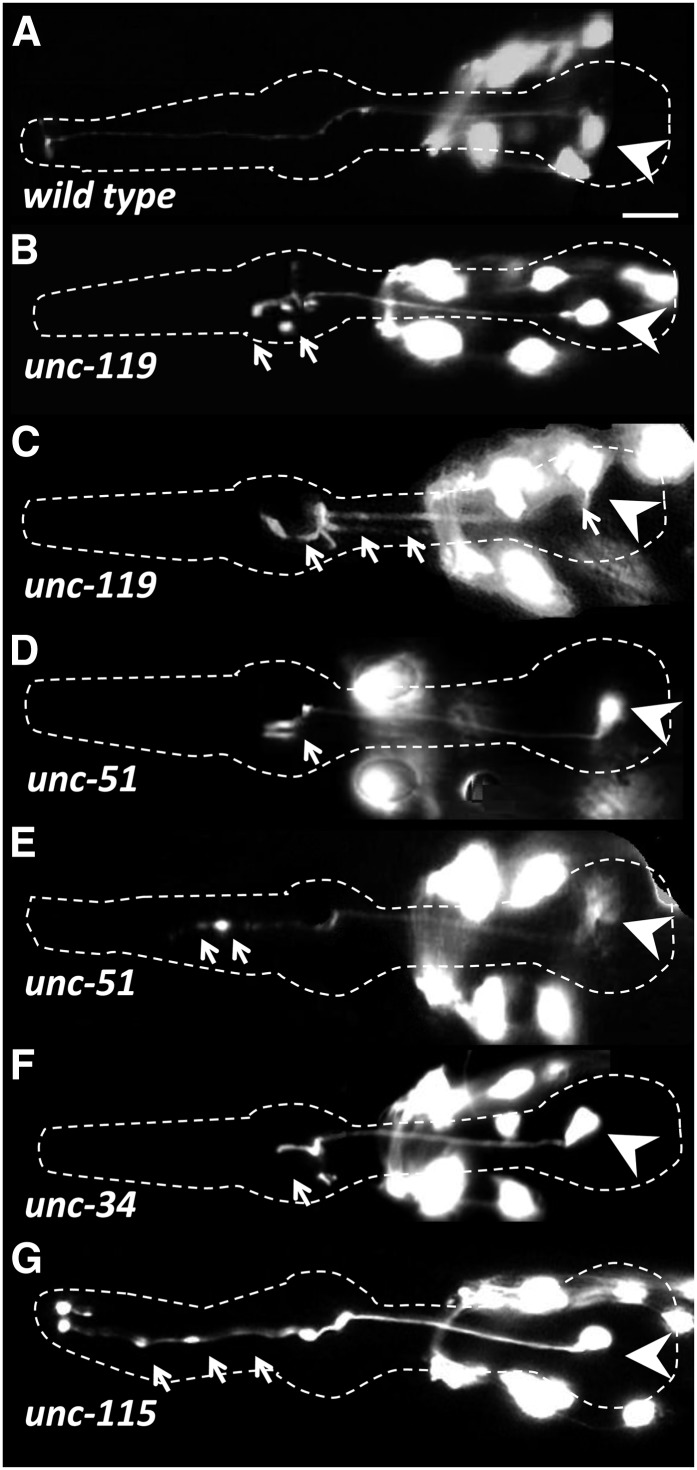
M1 axon abnormalities in growth cone defective mutants in L4 or adult stages. The M1 neurons express the *glr-2*::*gfp* reporter. The pharynx is outlined in each panel, arrowheads indicate the M1 cell body, and arrows highlight M1 defects. Other neurons visible in the figure are nonpharyngeal. Note that with the exception of *unc-119*, all mutations show incomplete penetrance (Figure 3). (A) Wild-type M1 neuron. (B) The *unc-119* young adult exhibits axon truncation in metacorpus with abnormal branching. (C) Older *unc-119* animals (L4 + 72 hr) exhibit axon truncations, bilateral branches in the isthmus, and abnormal branches from the cell body. The *unc-51* mutants exhibit axon truncations in metacorpus (D) or procorpus (E). (F) The *unc-34* mutants with a truncation of the M1 axon at the metacorpus accompanied with short branches. (G) The *unc-115* animals exhibit swelling of M1 axon throughout the procorpus and metacorpus. Scale bar in (A) = 10 µm.

The *unc-119* functions in axon elongation, guidance, branching, and fasciculation (Knobel *et al.* 2001; Maduro and Pilgrim 1995; Maduro *et al.* 2000; Materi and Pilgrim 2005). Human UNC119, which can rescue the *C. elegans* mutation, functions in receptor-associated activation of signal transduction and the transport of proteins, including myristolylated G protein alpha subunits and Src-type tyrosine kinases (Constantine *et al.* 2012; Gorska *et al.* 2004; Zhang *et al.* 2011). In 99% (n = 182) of *unc-119* mutants, the M1 axon was truncated in the metacorpus, often with abnormal branching and a failure to travel dorsally (Figure 3 and Figure 5, B and C). Additionally, M1 frequently exhibited ipsilateral outgrowths within the isthmus and ectopic outgrowths from the cell body (Figure 5C). The frequency of these latter phenotypes increased as animals aged from L4 to 3 days after L4 adults.

The *unc-51* encodes a conserved serine/threonine kinase involved in growth cone membrane dynamics (Asakura *et al.* 2010; Ogura *et al.* 2010; Ogura *et al.* 1994). In 50% (n = 270) of the *unc-51* mutants, the M1 axon was truncated either within the procorpus or in the anterior bulb, where it failed to reach the dorsal nerve cord and sometimes had ectopic branches (Figure 3 and Figure 5D). As revealed by GFP, defective axons also showed swelling along distal trajectory (Figure 5E). These structures may represent remnants of stalled growth cones, similar to those observed for the axons of pharyngeal M2 motor neurons in the metacorpus of *unc-51* mutants (Morck *et al.* 2003).

The *unc-34* encodes an Enabled/VASP actin-binding protein that controls growth cone filopodia formation in parallel to the Rac pathway (Gitai *et al.* 2003; Norris *et al.* 2009; Shakir *et al.* 2006; Withee *et al.* 2004; Yu *et al.* 2002). In 62% (n= 140) of *unc-34* mutants, the M1 axon prematurely terminated at the metacorpus or at the procorpus without bundling with the dorsal nerve cord (Figure 3 and Figure 5F). The abnormal axons that terminated in the metacorpus usually had short branches. We occasionally (5%) observed ipsilateral branches that ran in parallel to the original axon in the isthmus, in addition to short projections from the M1 cell body.

UNC-115/abLIM is an actin-binding protein that controls formation of lamellipodia and filopodia, and it is thought to be regulated by Rac (Demarco and Lundquist 2010; Struckhoff and Lundquist 2003; Yang and Lundquist 2005). The *unc-115* mutants did not show truncations but (20%; n = 130) exhibited minor defects of the M1 axon within the procorpus, such as swelling, similar to those found in *unc-51* (Figure 3 and Figure 5G). Mutants of genes that are regulated by Rac GTPases usually exhibit defects in axon outgrowth, likely as a result of impaired growth cone navigation (Bishop and Hall 2000; Gallo and Letourneau 1998; Yang and Lundquist 2005). Mutants for the Rac GTPases *ced-10* and *mig-2*, as well as the double mutant, exhibited similar M1 axon defects at the procorpus, albeit with much lower frequencies (2%) (Figure 3). Similarly, axonal extension mutants *unc-69*, *unc-73*, and *unc-76* exhibited minor low-penetrance defects of the M1 distal axon trajectory, such as short ectopic projections and swelling (Figure 3).

The defects we observed among all mutants known to have growth cone or axonal outgrowth phenotypes were restricted to the distal trajectory of M1. The initial proximal trajectory of M1 in the isthmus was always normal, suggesting that M1 uses two distinct mechanisms to build its trajectory.

### The gland cell may provide cues for growth cone–mediated guidance of the distal M1 axon

The M1 axon extension abnormalities scored in many of the mutants described phenocopied gland removal. However, neither these mutants (with the exception of *unc-119*) nor gland ablation resulted in 100% penetrance. Because we showed that gland ablation was up to 98% efficient, and because we used null or severe loss-of-function mutations, the incomplete penetrance of M1 defects suggests multiple mechanisms for distal M1 extension. To determine if the gland acts in parallel to the genes during M1 axon extension, we examined M1 axons in glandless *hlh-6*::*egl-1* worms that also carried mutations in those genes (Figure 3). If the gland and a particular gene act in parallel, redundant pathways, then removal of both would enhance the defects, although there would be no change if they acted together. Removal of the gland in mutant backgrounds showed no significant increase in the percentage of animals with M1 truncations (*P* ≥ 0.05 in all cases). More specifically, the axon truncation phenotype was changed from 50% to 57% for *unc-51* (n = 21) and from 62% to 69% for *unc-34* (n = 16, values are small because the strains grew very slowly). Gland removal in *unc-115* mutants, which have axon swelling rather than a truncation phenotype, exhibited 44% normal axons, similar to gland ablation alone (n = 16). These observations suggest that M1 uses the gland as a substrate for its outgrowth in the metacorpus and procorpus.

To test whether the M1 axon phenotype that we observed in *unc-51*, *unc-34*, and *unc-115* mutants was a secondary result of defects of the g1P gland projection, we examined gland shape in these mutants. The *unc-51* exhibited a weak effect on the g1P projection, causing minor swelling (5%; n = 20) (Supporting Information, Figure S1). The *unc-34* mutants showed a higher frequency of swelling and ectopic projections along the g1P process (42%; n = 20), with 3% premature terminations near the pharyngeal tip. The *unc-34* also affected other pharyngeal gland cells and the g1P cell body. The *unc-115* mutants showed some ectopic g1P projections and swelling (25%; n = 20). Overall, the g1P phenotypes in these mutants were much less severe than those of the M1 axon and, in particular, the gland projection always reached its final target (except in few *unc-34* mutants). These results suggest that M1 axon defects in these mutants may not be the secondary consequence of g1P defects. Additionally, these data suggest that the g1P gland does not need M1 to establish its projection in the procorpus.

### The distal trajectory of the M1 axon is only weakly affected by axon guidance mutations

The incomplete penetrance of the distal M1 axon phenotypes in the absence of the gland cell suggests that additional external guidance cues act redundantly with the g1P projection. To identify such genes, we tested mutations affecting the major axon guidance cues or their receptors. The four major guidance systems in both vertebrates and invertebrates include semaphorins and their plexin and neuropilin receptors (Castellani and Rougon 2002; Ginzburg *et al.* 2002; Tessier-Lavigne and Goodman 1996), Netrins and their DCC and UNC5 receptors (Hedgecock *et al.* 1990; Norris and Lundquist 2011; Shekarabi and Kennedy 2002), Slits and their Robo receptors (Brose *et al.* 1999; Hao *et al.* 2001), and Ephrins and their Eph receptors (Chin-Sang *et al.* 1999; George *et al.* 1998; Rodger *et al.* 2012).

**Figure 6:**
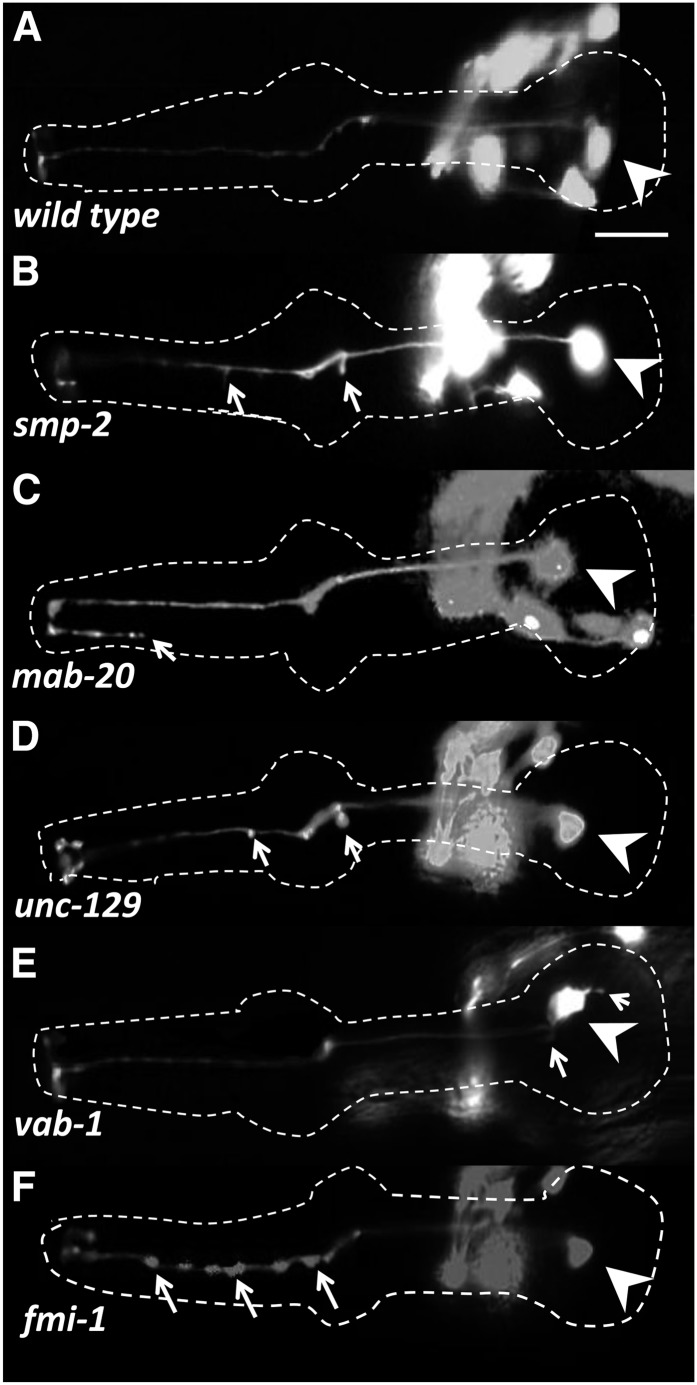
M1 axon phenotypes in L4 or young adult guidance and adhesion mutants. The M1 neuron expresses the *glr-2*::*gfp* reporter. The pharynx is outlined in each panel; arrowheads indicate the M1 cell body and arrows highlight M1 defects. Other neurons visible in the figure are nonpharyngeal cells that are part of the nerve ring. Note that penetrance is low in all cases (Figure 3). (A) Wild-type (this is the same image as shown in Figure 4A). (B) The *smp-2* exhibits small projections at the procorpus and metacorpus. (C) The *mab-20* shows overextensions of M1 terminal branches beyond their normal targets. (D) The *unc-129* has short projections and swelling of the M1 axon in the metacorpus. (E) The *vab-1* mutant has an apparently normal M1 axon, but one that exhibits abnormal minor branches from the cell body. (F) The *fmi-1* mutant shows abnormal swelling of the M1 axon in the procorpus. Scale bar in (A) = 10 µm.

*C. elegans* has two transmembrane semaphorins, SMP-1 and SMP-2, which signal through the PLX-1 plexin receptor (Castellani and Rougon 2002; Fujii *et al.* 2002; Tessier-Lavigne and Goodman 1996). Worms also have one secreted Semaphorin, MAB-20 (Roy *et al.* 2000), which signals through PLX-2 receptor (Ikegami *et al.* 2004; Nakao *et al.* 2007). In *smp-2* mutants, ectopic outgrowths were observed occasionally in the M1 axon within the metacorpus and in the procorpus, with a frequency of 6% (n = 180) (Figure 3 and Figure 6B). Although the M1 axon showed only 1% defects (ectopic projections in the metacorpus) in *smp-1*, these abnormalities were seen in 11% (n = 100) of the *smp-1smp-2* double mutants, suggesting that the two genes act redundantly (*P* < 0.05). The *plx-1* mutants exhibited defects (9%; n = 180) similar to those of *smp-1smp-2* double mutants. The *mab-20* and *plx-2* had low frequencies of ectopic projections in the metacorpus and also occasional posterior overextension (Figure 3 and Figure 6C).

UNC-6 is the *C. elegans* Netrin, and it has two receptors, UNC-40/DCC and UNC-5 (Chan *et al.* 1996; Culotti and Merz 1998; Hedgecock *et al.* 1990; Ishii *et al.* 1992; Livesey 1999). M1 was apparently normal in *unc-6* (n = 180 for each of two alleles) as well as in *unc-40* (n = 160) mutant backgrounds (Figure 3). The *unc-5* mutants exhibited minor ectopic projection from the M1 axon at the metacorpus, with a frequency of 4% (n = 178). This is the position where M1 moves dorsally into the dorsal pharyngeal nerve cord (Albertson and Thomson 1976).The TGF-beta family ligand UNC-129 physically interacts with UNC-5 to regulate cellular responses (MacNeil *et al.* 2009). The *unc-129* animals had ectopic projections similar to those observed in *unc-5*, albeit with a higher frequency of 12% (n = 130) (Figure 3 and Figure 6D).

SAX-3 acts with EVA-1 as Robo receptors for SLT-1, the only Slit protein in *C. elegans* (Fujisawa *et al.* 2007; Hao *et al.* 2001). We observed that mutants of *slt-1* and *sax-3* had little or no effect on the M1 axon (n = 100 each). VAB-1 is the sole Ephrin receptor in *C. elegans*, which receives signals from the Ephrins EFN-1, EFN-2, EFN-3, and EFN-4 (Arvanitis and Davy 2008; George *et al.* 1998). *vab-1* did not show M1 defects beyond a few ectopic projections from its cell body (2%; n = 100) (Figure 3 and Figure 6E).

We also tested components of Wnt signaling, which regulate migration of growth cones and cells along the *C. elegans* anterior–posterior axis (Fradkin *et al.* 2005; Hilliard and Bargmann 2006; Maro *et al.* 2009). Mutants for the Wnt ligand EGL-20 showed rare truncations of the M1 axon at the procorpus (1%; n = 100) (Figure 3). The M1 trajectory appeared normal on the loss of the Wnt receptor LIN-17 or the downstream target of Wnt signaling PRY-1. We also tested *egl-15*, which encodes the only worm fibroblast growth factor receptor and plays a role in axon guidance and fasciculation in the ventral nerve cord (Bulow *et al.* 2004). We found 3% of M1 distal axons (n = 50) had ectopic swelling or branching (Figure 3).

None of the null or strong loss of function mutants we used showed defects in initial M1 axon outgrowth or resulted in distal M1 truncations. These results suggest that tested guidance cues play minor roles in M1 axon outgrowth. It is possible that these pathways/genes act redundantly to guide the M1 axon. We tested double mutants, including *plx-1*; *unc-129* (whose individual mutants had the highest penetrance among guidance genes), *unc-5*; *unc-129*, *unc-51*; *unc-129*, *unc-51*; *unc-5*, *unc-115*; *egl-15*, and *vab-1/+*; *sax-3* (*vab-1*; *sax-3* homozygotes arrest before the stage of *glr-2*::*gfp* expression) (Ghenea *et al.* 2005). All combinations were essentially identical to the allele with the highest penetrance (Figure 3) (*P* > 0.05 in all cases), arguing against redundancy between the tested pathways.

### The distal trajectory of the M1 axon is only weakly affected by known adhesion mutants

Because distal M1 axon outgrowth is influenced by loss of either glands or genes with axonal outgrowth functions, we examined adhesion molecules that the M1 axon might use to fasciculate to the g1P gland projection. We screened candidates known to be expressed in the pharynx, glands, or neurons. The *C. elegans* flamingo-like cadherin, *fmi-1*, controls axon pathfinding, fasciculation, and synapse morphology (Najarro *et al.* 2012; Steimel *et al.* 2010). The *fmi-1* mutants exhibited occasional bright GFP swellings of the M1 axon in the procorpus (7%; n = 100), which could be fasciculation defects or aberrant synapses (Figure 3 and Figure 6F). We found no defects in mutants of the Fat-like cadherin CDH-4, which controls axon fasciculation and cell migration (Schmitz *et al.* 2008). The redundantly acting IgCAMs RIG-3 and RIG-5 are expressed in M1 and g1P, respectively (Schwarz *et al.* 2009). Thus, we tested the octuple mutant *rig-5*; *rig-6*; *rig-4*; *rig-1wrk-1rig-3syg-1ncam-1* (Schwarz *et al.* 2009) but observed no M1 defects (Figure 3).

### New mutations with abnormalities in M1 guidance and development

**Figure 7:**
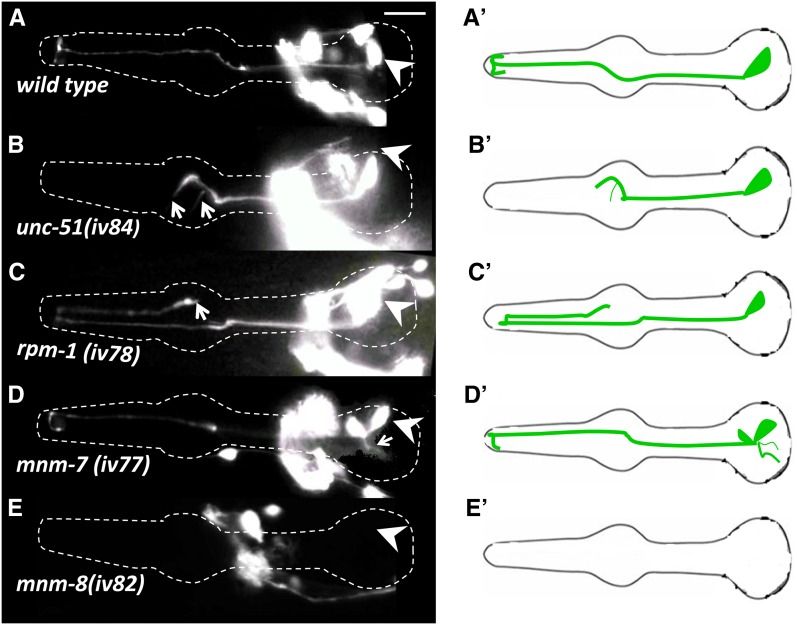
Phenotypes of mutants isolated from the forward genetic screen visualized in L4 or young adults. M1 is marked with *glr-2*::*gfp*. Arrowheads indicate cell bodies and arrows denote defects, which are incompletely penetrant in all cases (Figure 3). Other neurons visible in the figure are part of the nonpharyngeal cells in the nerve ring. (A′–E′) Cartoons for each phenotypic class. (A) Wild-type. (B) The *unc-51(iv84)* shows axon truncation and an ectopic branch. (C) The *rpm-1(iv78)* results in overextension of M1 terminal branches beyond their normal targets. (D) The *mnm-7(iv77)* shows ectopic branching from the cell body. (E) The *mnm-8(iv82)* shows a 95% penetrant M1 missing phenotype, whereas the other neurons that express the *glr-2*::*gfp* reporter are still present. Scale bar in (A) = 10 µm.

Because the M1 axon defects showed incomplete penetrance in all situations that we examined (with the exception of *unc-119*), M1 axon extension and guidance may involve novel genes. Thus, we searched for viable mutants with M1 morphology or developmental defects using the integrated *glr-2*::*gfp* strain. In a screen of 5000 mutagenized haploid genomes, nine mutations were identified that fell into four phenotypic classes: axon truncation; axon overextension; ectopic branches at the cell body; and M1 absent (Figure 3 and Figure 7). None affected the initial outgrowth of the M1 axon through the isthmus.

The truncation mutation *iv84* exhibited premature termination of the M1 axon in the procorpus or the metacorpus in 50% of animals (n= 200) (Figure 3). This mutant also has a severe Unc phenotype. The *iv84* was mapped to the right arm of chromosome V and failed to complement *unc-51(e268)*. Identification of *iv84* in a gene known to be required for M1 axon extension serves as proof-of-principle for the effectiveness of the screen.

In the axon overextension class, one or both of the terminal posteriorly facing M1 axon branches overshoots the final target, extending further posteriorly, sometimes as far as the anterior bulb (Figure 3 and Figure 7B). The *iv78*, *iv79*, *iv80*, and *iv81* mapped to the middle of chromosome V and failed to complement each other, as well as alleles of *rpm-1*. RPM-1 (regulator of presynaptic morphology-1) is an E3 ubiquitin ligase that contains a guanine nucleotide exchange factor domain. RPM-1 is a member of the conserved Pam/Highwire (PHR) protein family, which regulates axon termination and synaptogenesis (D’Souza *et al.* 2005; Li *et al.* 2008; Schaefer *et al.* 2000; Wan *et al.* 2000; Zhen *et al.* 2000). As expected, the previously identified alleles, *rpm-1(js317)* and *rpm-1(ur299*), showed the same axon phenotypes (Figure 3). Thus, the formation of synapses on pm1 and pm2 may have a role in marking the termination of M1 axon extension. Another overextension mutation, *iv88*, was also Unc, and this mutation mapped to the middle of chromosome IV. There were no obvious candidates in this region, and we assigned it the gene name *mnm-6* (M neuron morphology abnormal) (Morck *et al.* 2003).

The branching cell body mutations *iv77* and *iv90* have short, abnormal outgrowths from the M1 cell body and occasional axon overextensions (similar to the overextension class) (Figure 3 and Figure 7D). Nonpharyngeal neurons in the nerve ring that express *glr-2*::*gfp* are also affected, suggesting a general axon guidance defect. These two mutations failed to complement and both mapped to the right arm of chromosome III. There are no obvious candidates in the 2-cM region where they mapped. We assigned them the gene name *mnm-7*.

In the M1 missing mutation *iv82*, pharyngeal *glr-2*::*gfp* was absent in 95% of animals, but the transgene still was expressed in nonpharyngeal neurons in the nerve ring and ventral nerve cord (Figure 3 and Figure 7E). M1 was absent at high frequencies at all stages at which we could visualize the neuron (*i.e.*, as early as three-fold). This mutant also retained a fully penetrant protruding vulva phenotype after six outcrosses. The *iv82* mapped to the right arm of chromosome I. The Wnt pathway gene *pry-1* (Korswagen *et al.* 2002; Maloof *et al.* 1999) maps to this region, but M1 is normal in both *pry-1/pry-1* and *pry-1/iv82*. The *iv82* has been assigned the gene name *mnm-8*.

The absence of the M1 neuron in *mnm-8* could result from transformation of M1 into its sister MSpaapaap, which normally undergoes programmed cell death (Sulston *et al.* 1983). In *ced-3* mutants that block apoptosis (Lettre and Hengartner 2006), we observed an additional neuronal-like cell, indicating that the undead M1 sister probably adopts an neuronal-like fate. However, in *mnm-8*; *ced-3*, we observed neither cell, indicating that *mnm-8* affects both M1 and its putative sister rather than inducing apoptosis by transforming M1 into its dying sister. Thus, *mnm-8* may affect either the generation of M1 or its ability to express the *glr-2*::*gfp* reporter (which is still expressed in other nonpharyngeal neurons) (Figure 7E).

## Discussion

In this work, we explored the development of the *C. elegans* M1 motor neuron, which extends almost the entire length of the pharynx, as a model of how axons are extended within a simple structure, the pharynx. Our results indicate that axon extension in the pharynx appears to be robust and its proximal outgrowth and distal outgrowth are mediated by two different mechanisms.

### The proximal and distal phases of M1 axon extension use distinct mechanisms

**Figure 8:**
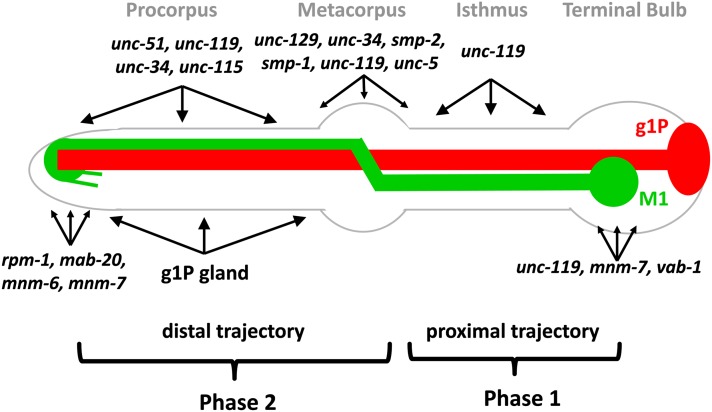
Model for M1 axon guidance. M1 is shown in green and g1P is shown in red. M1 development is divided into two steps. During phase 1, M1 builds its proximal trajectory independent of genes affecting growth cones. In phase 2, M1 builds its distal trajectory, which is affected in growth cone–defective mutants and loss of the g1P cell. The regions where M1 axon phenotypes occur are shown for each gene. These genes are categorized in groups, which act in metacorpus, procorpus, and or at the anterior pharyngeal tip, where the axon terminates. The *unc-119* (which also acts for distal extension), *mnm-7*, and *vab-1* are necessary for preventing ectopic branching in the isthmus or terminal bulb, rather than axon extension in those regions.

We demonstrated that the extension of the M1 axon occurs in two distinct phases (Figure 8). The first (proximal) phase through the isthmus was normal under all of the conditions we tested and not affected by gland ablation, mutations that impair growth cone function, axonal outgrowth, or mutations in genes known to affect axon guidance or cell adhesion. Moreover, none of the mutants that we isolated from our screen for M1 defects exhibited abnormalities during the proximal phase of axon extension. In contrast, the subsequent distal phase of M1 axon extension through the metacorpus and procorpus was often abnormal after gland ablation, with mutations affecting growth cones and axonal outgrowth, and, to a lesser extent, with mutations affecting axon guidance.

The proximal extension of the M1 axon (Figure 3 and Figure 5) appears similar to that described for outgrowth of the bilateral M2 and NSM pharyngeal neurons (Axang *et al.* 2008; Morck *et al.* 2003; Pilon 2008). The M2 neuron growth cones appear only after the axon has extended through the nascent isthmus (Rauthan *et al.* 2007). Pilon *et al.* (Axang *et al.* 2008; Morck *et al.* 2003; Pilon 2008) proposed that elongation of M2 axons and the minor processes of the NSM pharyngeal neurons are analogous to the “fishing line” model (Bray 1984) by attachment to a neighboring cell. The cells are later moved to opposite sides of the isthmus during pharyngeal morphogenesis, elongating the axon. During *C. elegans* embryogenesis, the pharynx primordium elongates from a ball-shaped cell mass to the bilobed shape of the mature pharynx as the embryo as a whole transforms from a spheroid to a tube (Portereiko and Mango 2001). This elongation likely provides the force for initial separation of the cell body from its axonal anchor point. Our results demonstrated that the M1 proximal trajectory is not affected by mutations in genes with axonal outgrowth phenotypes, suggesting that M1 may use a similar mechanism to extend through the isthmus. However, we could not observe outgrowth directly because of the lack of a specific M1 marker expressed before axon elongation.

### The pharyngeal gland g1P guides the distal trajectory of the M1 axon

M1 is the first studied pharyngeal neuron that extends its axon into the procorpus, which occurs during the second (distal) portion of its outgrowth. The position of the non-neuronal pharyngeal g1P gland projection adjacent to the M1 axon in the procorpus (Albertson and Thomson 1976) prompted us to ask whether M1 uses g1P to establish its distal trajectory (Figure 1). Genetic ablation of the pharyngeal gland cells caused defects in the distal portion of the M1 axon, including truncations, abnormal branches, and abnormal trajectories (Figure 2 and Figure 3). Abnormalities were never observed within the isthmus, where M1 does not contact the g1P projection. It is clear from our time course analysis that we killed the gland cells before g1P began extending its projection (Figure 4), and this occurred before the earliest time that we could observe the M1 axon. The early induction of gland cell death and the appearance of M1 axon defects as soon as the neuron became visible argue against the possibility that phenotypes were caused by maintenance rather than initial guidance.

It is formally possible that M1 is influenced by gland cells other than g1P, which were also killed by *hlh-6*::*egl-1* (data not shown). However, M1 is never in contact with the other gland cells, which do not extend projections beyond the isthmus into the region of interest (Albertson and Thomson 1976; Smit *et al.* 2008).

It is notable that we did not observe a reciprocal effect of M1 on the g1P projection. In most mutants that showed M1 extension defects, we never observed g1P truncation, and even minor structural defects of the g1P projection (*e.g.*, ectopic branches or minor misroutes) were much less severe than for M1. Importantly, in mutants with frequent M1 truncations, the g1P projection was always able to extend fully (Figure S1). In *mnm-8(iv82)*, the g1P projection never failed to reach its final target, although an M1-like (*glr-2*::*gfp* expressing) neuron was not present in 95% of animals (data not shown). Together, these data indicate that the g1P gland does not need the M1 axon to establish its projection.

The incomplete penetrance of the M1 defects in gland-ablated animals could reflect redundancy between guidance by g1P and other cells that bundle with M1 in the pharyngeal dorsal nerve cord. However, ablation of such a cell, the I3 interneuron, alone or in combination with the gland cell ablation, did not have additive effects on M1 guidance.

One possibility is that g1P acts as a pioneer neuron to which the M1 axon fasciculate. However, we failed to observe the M1 axon defects in multiple adhesion molecule mutants, including *fmi-1* and mutations of other IgCAM genes that are expressed in the pharynx and that could potentially mediate the M1/g1P fasciculation (Figure 3). Thus, multiple cells or adhesive molecules may act redundantly to guide the M1 distal axon. Alternatively, g1P may act as a guidepost at the metacorpus to attract the M1 axon toward the dorsal nerve cord, after which the M1 axon is guided by other mechanisms. This role is analogous to that of the M2 neurons’ sister M3, which is thought to send an instructive signal to guide M2 growth cones (Pilon 2008; Rauthan *et al.* 2007). Another possibility is that g1P may function in a role usually performed by neuronal supporting cells such as glia. Many studies have demonstrated that glial cells mark choice points for axon growth (Auld 1999; Hidalgo 2003; Hidalgo and Booth 2000; McDermott *et al.* 2005; Puche and Shipley 2001; Silver *et al.* 1982; Spindler *et al.* 2009), and this also occurs in *C. elegans* (Bacaj *et al.* 2008; Heiman and Shaham 2009; Oikonomou and Shaham 2011; Yoshimura *et al.* 2008).

### Genes that function in axon extension guide the distal trajectory of the M1 axon

In contrast to the first phase of outgrowth, M1 axon extension within the isthmus was affected by mutations known to affect growth cone function and axon extension, including *unc-34*, *unc-51*, *unc-115*, and *unc-119*. However, defects were not fully penetrant in any of the conditions we examined (with the exception of *unc-119* mutants), suggesting that M1 uses multiple or overlapping mechanisms to establish its distal trajectory. It is possible that *unc-34*, *unc-51*, and *unc-115* act autonomously within the M1 cell. These genes are normally expressed in worm neurons during their development and have been demonstrated to act cell-autonomously during the outgrowth of many neurons (Fleming *et al.* 2010; Knobel *et al.* 2001; Lai and Garriga 2004; Lundquist *et al.* 1998; Sheffield *et al.* 2007). However, there are examples of *unc-51* acting nonautonomously during axon guidance (Ogura *et al.* 2010). If *unc-34*, *unc-51*, or *unc-115* were acting nonautonomously for M1 extension, the most likely focus would be the neighboring I3 interneuron or the g1P gland. However, ablation of I3 had no effect on the M1 cell (Figure 3). It also is unlikely that M1 phenotypes stem from the genes acting within g1P rather than M1 because *unc-34*, *unc-51*, and *unc-115* mutations had, at best, weak effects on g1P and rarely prevented full extension of the g1P process (Figure S1). Furthermore, the g1P projection does not extend by a process similar to axonal outgrowth, but rather is anchored in the anterior of the pharynx and is then drawn out as the cell body migrates posteriorly (J. Kormish and J. Gaudet, unpublished data). It is possible that these genes influence signals that M1 receives from adjacent muscle cells, but it seems unlikely that each of the three genes, with well-characterized axonal outgrowth functions, are all acting in a novel way. The most parsimonious model shows that most, if not all, of the genes act within M1.

Ablating the gland cells in *unc-34*, *unc-51*, and *unc-115* had, at best, weakly additive effects (Figure 3). This suggests that the mutations and g1P act in the same, rather than different, pathways, for example, an M1 growth cone could track along the g1P extension.

The *unc-119* differs from the other tested genes and gland ablation in showing near-complete penetrance. There are examples of UNC-119 acting either cell-autonomously or nonautonomously in *C. elegans* (Knobel *et al.* 2001; Materi and Pilgrim 2005). Whether *unc-119* influences the M1 phenotype by acting in M1 or other pharyngeal cells is unknown.

### Genes known to affect guidance signaling pathways have modest roles in M1 axon extension

We examined mutations in genes that encode axon guidance ligands and receptors to find those that might act in parallel to the g1P in guiding distal M1 axon outgrowth. None of these genes showed a profound effect on M1 axon that was similar to the defects caused by gland ablation or mutations in genes with growth cone or axonal extension functions. For example, mutants for the dorsal/ventral guidance pathway (Hedgecock *et al.* 1990; Ishii *et al.* 1992) had weak effects in which M1 deflects toward the dorsal nerve cord. Mutants of the Netrin receptor gene, *unc-5*, and the TGF-beta family ligand, *unc-129* (MacNeil *et al.* 2009), exhibited 4% and 12%, respectively, short ectopic projections in metacorpus (Figure 3). UNC-129 interacts with UNC-5 to regulate cellular responses to UNC-6 (MacNeil *et al.* 2009), but *unc-5*; *unc-129* double mutation had little additive effect on M1 (Figure 3). Two *unc-6*/Netrin alleles resulted in only 1% defects, suggesting that if there is a minor role for UNC-5, it could be independent of UNC-6. For the Semaphorin system, *plx-1* or the *smp-1smp-2* double mutant had ∼10% short ectopic branching in the metacorpus, with *mab-20* and *plx-2* showing weaker effects (Figure 3). Finally, SAX-3/Robo receptor of the Slit/Robo pathway had no effect on M1 guidance. We were unable to detect redundancy between the guidance systems among *plx-1*; *unc-129*, *unc-5*; *unc-129*, *unc-51*; *unc-129*, *unc-51*; *unc-5*, *unc-115*; *egl-15*, or *vab-1/+*; *sax-3* (Figure 3).

### Different pharyngeal neurons use overlapping and distinct mechanisms to extend their axons

M1 both shares and has unique axonal elongation mechanisms with the other two pharyngeal neurons that have been studies in detail, M2 and NSM (Axang *et al.* 2008; Morck *et al.* 2003; Rauthan *et al.* 2007; Pilon 2008). Both M1 and M2 are influenced by interactions with another cell (g1P and M3, respectively). The minor process of NSM and the proximal trajectories of the M1 and M2 axons extend independent of genes that affect axon extension, such as *unc-34*, *unc-51*, *unc-115*, and *unc-119*. The axonal extension phase that does depend on these genes differs between the three types of neurons, for example, NSM extension does not require *unc-119*, whereas M1 and M2 require *unc-119* to suppress ectopic branching. M1 differs from M2 and the major NSM projections in showing only weak *unc-69*, *unc-73*, and *unc-76* phenotypes. The most dramatic difference is that M1 shows only weak phenotypes for mutations in the major guidance pathways. Whereas both M1 and NSM show phenotypes for Semaphorin and *unc-129* mutations, the defects were much weaker for M1. M1 also differs from the other two neurons by not showing dependence on the Netrin and Slit systems. A similarity between the three neurons is that none responded to Ephrin mutations. Extension phenotypes of the three types of pharyngeal neurons and, indeed, for most *C. elegans* neurons, nearly always showed incompletely penetrant defects, highlighting the robustness of the process.

### New mutations affecting M1

Because all situations tested resulted in incomplete penetrance for distal M1 extension, novel molecules might be involved in M1 guidance. We thus performed a forward genetic screen for abnormal M1 morphology using the integrated *glr-2*::*gfp* maker and identified nine mutations resulting in axon truncation, abnormal branching, or termination defects. This screen isolated new *unc-51* alleles (demonstrating the utility of the screen) and *rpm-1*. We also identified mutations in three novel genes, *mnm-6*, *mnm-7*, and *mnm-8*.

The RPM-1 protein functions in synaptogenesis and axon termination in different organisms (D’Souza *et al.* 2005; Li *et al.* 2008; Schaefer *et al.* 2000; Wan *et al.* 2000; Zhen *et al.* 2000). In *C. elegans*, RPM-1 acts cell-autonomously in the PLM mechanosensory neuron and DA/DB motorneurons to regulate axon termination, guidance, and synapse formation (Grill *et al.* 2007; Li *et al.* 2008; Trujillo *et al.* 2010; Tulgren *et al.* 2011). Consistent with these observations, the *rpm-1* mutants, including both existent alleles and those isolated in our screen, exhibited failure of the M1 terminal process to stop at their final destination. Rather, they extend posteriorly, overshooting their normal targets. This is the first case to describe a role for *rpm-1* in the pharyngeal nervous system.

The Unc phenotype of the *mnm-6* overextension mutant may indicate that the gene functions in guiding other neurons. Because *mnm-6* shows a stronger phenotype than any of the axon guidance–null mutants we tested, it may function independently of the guidance pathways that we tested. The *mnm-7* also has general effects on axon guidance because other *glr-2*::*gfp* neurons outside the pharynx show overextension and abnormal branching. The *mnm-8* results in the absence of M1 neuron in 95% of animals, whereas other *glr-2*::*gfp*–expressing neurons were present. The *mnm-8* likely affects some aspect of M1 specification or differentiation that leads to absence of the *glr-2*::*gfp* expression rather than transforming M1 to the fate of its sister, which normally undergoes programmed cell death (Sulston *et al.* 1983). It will be interesting to see whether any other neurons that emerge from the pharyngeal primordium differentiate in an *mnm-8*–dependent manner.

A simple model for M1 axon development involves two phases (Figure 8). In the first phase, M1 builds its proximal trajectory using mechanisms distinct from those used during the rest of the axon journey and may be coupled to elongation of the pharynx as a whole. In the second phase, M1 uses multiple mechanisms to establish its distal trajectory, likely including growth cones and cues from the non-neuronal g1P gland cell. Incomplete penetrance under all the tested conditions suggests that this phase may use highly redundant pathways, involve novel mechanisms, or depend on mutations with lethal phenotypes. Alternatively, the route of the M1 axon may be physically constrained by surrounding pharyngeal tissues such that the axon sometimes extends properly when normal guidance cues using g1P or the growth cone are compromised. The M1 system is a compelling model for axon guidance in the pharynx with similarities and differences from previously described models such as M2 and NSM neurons (Axang *et al.* 2008; Morck *et al.* 2003). Even though the pharynx represents a closed and limited environment of only 80 cells (Albertson and Thomson 1976), axon guidance in this simple structure is nevertheless a complex process.

## Supplementary Material

Supporting Information
