# Supplementary material for: Extension of the Caenorhabditis elegans Pharyngeal M1 Neuron Axon Is Regulated by Multiple Mechanisms
Source: G3 (Bethesda). 2013 Nov 1;3(11):2015–29. doi: 10.1534/g3.113.008466 (PMC3815062; doi:10.1534/g3.113.008466)
Supplement: Supporting Information [file supp_3_11_2015__index.html]

Extension of the Caenorhabditis elegans Pharyngeal M1 Neuron Axon Is Regulated by Multiple Mechanisms — Supporting Information 

# Extension of the *Caenorhabditis elegans* Pharyngeal M1 Neuron Axon Is Regulated by Multiple Mechanisms

## Supporting Information for Refai *et al.*, 2013

**Files in this Data Supplement:**

- Figure S1 - The effect of growth cone mutations on the g1P gland cell projection. (PDF, 490 KB)
