# Supplementary material for: Extension of the Caenorhabditis elegans Pharyngeal M1 Neuron Axon Is Regulated by Multiple Mechanisms
Source: G3 (Bethesda). 2013 Nov 1;3(11):2015–29. doi: 10.1534/g3.113.008466 (PMC3815062; doi:10.1534/g3.113.008466)
Supplement: Supporting Information [file supp_g3.113.008466_008466SI.pdf]

G3: Genes | Genomes | Genetics

Extension of the *Caenorhabditis elegans* pharyngeal M1 neuron axon is regulated by multiple mechanisms

Osama Refai, Patricia Rohs, Paul E. Mains and Jeb Gaudet

Department of Biochemistry and Molecular Biology
Alberta Children's Hospital Research Institute
University of Calgary
Calgary, Alberta T2N 4N1
Canada

DOI: 10.1534/g3.113.008466

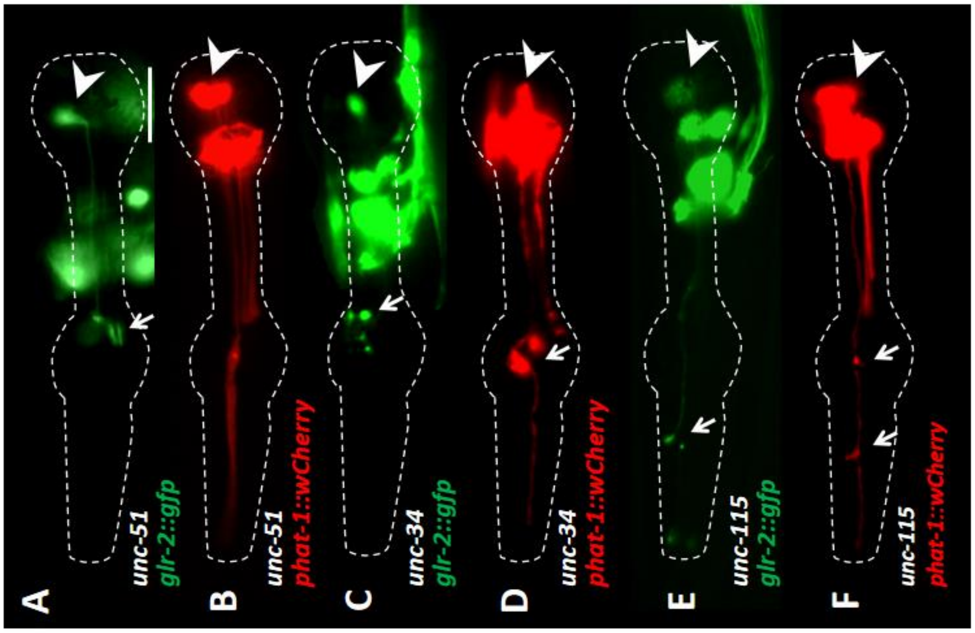

Figure S1 The effect of growth cone mutations on the g1P gland cell projection. g1P is labelled by *phat-1::wCherry* (red) and the M1 neuron is labelled by *glr-2::gfp* (green). The g1P projection often shows abnormalities in the mutants (arrows), but unlike the M1 axon in these mutants, always extends the length of the pharynx. The respective cell bodies are indicated by arrowheads and abnormalities by arrows. (A,B) *unc-51*. (C,D) *unc-34.* (E,F) *unc-115.* Scale bar in (A) = 10 µm.
